# A research tool for measuring non-participation of older people in research on digital health

**DOI:** 10.1186/s12889-019-7830-x

**Published:** 2019-11-08

**Authors:** Arianna Poli, Susanne Kelfve, Andreas Motel-Klingebiel

**Affiliations:** 1 Division Ageing and Social Change (ASC), Linköping University, Kungsgatan 40, 601 74 Norrköping, Sweden; 2 Aging Research Center (ARC), Karolinska Institutet & Stockholm University, Gävlegatan 16, 113 30 Stockholm, Sweden

**Keywords:** Digital health, Old age inequality, Social exclusion, Digitalisation, Recruitment, Self-selection, Non-participation

## Abstract

**Background:**

Healthcare services are being increasingly digitalised in European countries. However, in studies evaluating digital health technology, some people are less likely to participate than others, e.g. those who are older, those with a lower level of education and those with poorer digital skills. Such non-participation in research – deriving from the processes of non-recruitment of targeted individuals and self-selection – can be a driver of old-age exclusion from new digital health technologies. We aim to introduce, discuss and test an instrument to measure non-participation in digital health studies, in particular, the process of self-selection.

**Methods:**

Based on a review of the relevant literature, we designed an instrument – the NPART survey questionnaire – for the analysis of self-selection, covering five thematic areas: socioeconomic factors, self-rated health and subjective overall quality of life, social participation, time resources, and digital skills and use of technology. The instrument was piloted on 70 older study persons in Sweden, approached during the recruitment process for a trial study.

**Results:**

Results indicated that participants, as compared to decliners, were on average slightly younger and more educated, and reported better memory, higher social participation, and higher familiarity with and greater use of digital technologies. Overall, the survey questionnaire was able to discriminate between participants and decliners on the key aspects investigated, along the lines of the relevant literature.

**Conclusions:**

The NPART survey questionnaire can be applied to characterise non-participation in digital health research, in particular, the process of self-selection. It helps to identify underrepresented groups and their needs. Data generated from such an investigation, combined with hospital registry data on non-recruitment, allows for the implementation of improved sampling strategies, e.g. focused recruitment of underrepresented groups, and for the post hoc adjustment of results generated from biased samples, e.g. weighting procedures.

## Background

More and more studies are being conducted to evaluate new digital health technologies that might be incorporated into the provision of healthcare services and also meet the needs of older people, among other user groups [1]. The results are promising and show that digital health technologies can bring benefits at individual and organisational levels [1–3]. However, such results are often obtained based on evaluations conducted with selected groups of individuals who are typically younger, are better educated and have higher digital skills compared with older end-users [4–7]. In many cases, older end-users are still less likely to be digitally engaged and experienced [8], and hence risk being disadvantaged by such digital shift.

The overall aim of the present paper is to introduce, discuss and test an instrument to measure non-participation in digital health studies. Applying such an instrument can generate information for improved sampling strategies in digital health studies and provide data to enhance the quality of research outcomes by post hoc adjustments of results generated from biased samples.

First, we explore the nature of non-participation and its relevance in digital health research. Second, we discuss the conceptualisation of an approach to study non-participation and the two processes behind it – non-recruitment and self-selection. Third, we introduce an instrument for the analysis of self-selection. Fourth, we present the results of a pilot study of the instrument. Finally, we consider its use and applicability and knowledge generated by it.

### Digitalisation of healthcare services

European national healthcare systems have started to move towards a provision of healthcare which also includes services offered by digital means, such as scheduling medical appointments on the web, redirecting patients to web portals for health information and education, communicating remotely with care providers (e.g., e-Prescription), accessing personal health records (PHRs) online, and remote monitoring of patients’ health (e.g., telemedicine, telehealth) [9, 10]. This trend is driven by broader policy strategies that foster the creation of a digital society as a key to economic growth and sustainability [11] and has been decisively influenced by the accumulation of available technological advances [12].

Older people are expected to embrace such technological shifts in healthcare just as much as other age groups [12]. Several studies have been conducted to explore the potential benefits resulting from incorporating digital health technologies, at both individual and organisational levels, and some potentials have been recognised [1, 2].

### Digital inequalities among older people

Although digital health incorporation appears to be very promising, the way individuals embrace the opportunities that digitalisation offers can vary substantially [13], as do the benefits each individual gains from it. Even though the proportion of older people using technology and the Internet has increased significantly in recent years, a high proportion of older people are still not digital users or experienced digital users [14, 15].

On the one hand, the lower engagement of older people with digital technologies may reflect several different aspects, ranging from a lack of skills and material resources [16] – which is often related to their sociodemographic and socioeconomic backgrounds [17, 18] – and a lack of exposure to technology over their lifetimes [8, 18], to intentionally choosing not to use new digital technologies [19]. Moreover, physical and cognitive impairments can reduce the use of technology among older people [20, 21]. Focusing specifically on digital health technologies, those who, for example, are in the oldest age groups, have a lower socioeconomic status, have poorer digital skills and do not perceive digital health as useful in their everyday life, are less likely to engage with such services [20, 22–24].

Digital health research can be a driver of exclusion if some groups of people, such as more vulnerable groups, are underrepresented and their specific needs are neglected. Such limitations of digital health research can lead to these groups of people being denied the opportunity to benefit from new digital technologies [5, 25].

### Non-participation as a driver of inequalities

When conducting health-related research evaluating new digital health technologies, some individuals are less likely to be involved than others. Such a selection occurs during the process of recruiting participants, where a combination of study requirements and individual decisions to participate makes it challenging to recruit a group of study participants which is large and representative enough to reflect the heterogeneity of the population of interest [6, 25–29].

Health-related research often suffers from non-participation which can bias the results of the research and lead to the wrong conclusions. Non-recruitment derives from the non-recruitment of targeted individuals [30–32] and individual decisions not to participate [6, 26, 28]. As a result, many studies involve highly selected samples of individuals [5, 25] which do not reflect the overall population of interest.

Study participants and non-participants can differ considerably, with the former group typically being younger and in better health, and having higher digital skills and better socioeconomic conditions [7, 33].

The differences between participants and non-participants indicate that these two groups can differ in terms of their needs. Therefore, the underrepresentation of some groups of people in the research implies that some of the needs and interests of the target population are neglected and research results are biased.

#### Process 1: non-recruitment

**Fig. 1:**
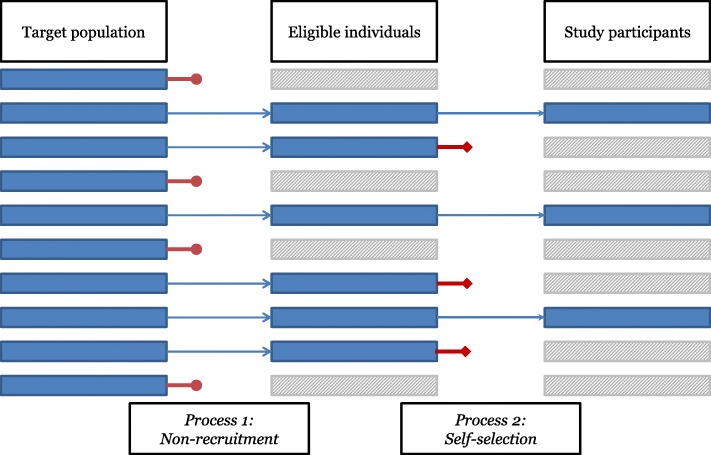
Processes behind non-participation in the process of recruiting study participants. Legend: Non-participation results from two processes: (1) non-recruitment and (2) self-selection. (1) the non-recruitment process distinguishes eligible individuals from non-recruited individuals, within the target population, and (2) the self-selection process distinguishes study participants from those who decline to participate, among the eligible individuals

In the process of recruiting study participants, medical conditions and scientific reasons can guide evaluations of eligible patients for the study and, thus, the non-recruitment of others. This represents the first process of selection, namely non-recruitment, which distinguishes eligible individuals from non-recruited individuals (Fig. 1). This often includes the non-recruitment of, for example, the oldest old, people with hearing and sight impairments, people with cognitive impairments and dementia, people with multiple comorbidities and people with poor medical compliance, who are deemed to be more likely to drop-out from a research study [30–32, 34–36].

#### Process 2: self-selection

In addition to non-recruitment, a further key mechanism of selection occurs for those people who have been deemed eligible and are thus invited to take part in the research: the self-selection (Fig. 1). Self-selection is based on the individual decision to participate in the study and distinguishes consenting people (i.e., the final group of study participants) from those who decline to participate.

Self-selection has often been reported in health-related and digital health research. Among patients with chronic obstructive pulmonary disease invited to participate in a controlled telemedicine trial, more than 50% declined the invitation to participate [26]. More than 75% of eligible patients declined to participate in a study testing homecare telemedicine in primary care [28], while the 80% of invited older patients to a randomised controlled trial (RCT) of home telecare did not agree to participate in the research [6].

Several predictors of self-selection in health-related research are discussed in the literature, such as socioeconomic conditions, health and quality of life (QOL), social participation and time resources. Research participants are likely to be younger than those who decline to participate [6, 26, 37–41]. Decliners have also been found to have lower levels of education [7, 41, 42] and lower QOL [42], worse health [37, 38, 41, 43–45], more cognitive impairment [45] and higher levels of perceived social support [41] compared to participants. The role of gender is less clear. Some studies show that men are more likely than women to participate in health-related research [7, 26, 39, 40], while others demonstrate the opposite [46] or no [38] effect. Lack of time and competing tasks have been described as further reasons for declining participation in health-related research [33, 38, 47–49], and in some cases the lack of time was due to care-related activities [37]. Participants, on the other hand, participated in the research because they hoped to improve their health and benefit from more care and support [50], and to contribute to science and society [50, 51].

Reasons for not participating when invited to participate in an evaluation of digital health technologies can often be attributed to technology, such as a lack of access to the Internet and computer devices [33], a lack of skills or of familiarity with computer and Internet use [33, 49, 52, 53], or a lack of perceived usefulness of technology (e.g., the belief they could not get any additional benefit from the technology) [28, 49].

The two processes of non-participation – non-recruitment and self-selection – work independently, but together they determine the selectivity in a sample. This affects the appropriateness of the results regarding accessibility, usability and suitability of new digital solutions. Outcomes might differ between underrepresented people and those who are more likely to participate. These considerations call for the development of an innovative research approach to understand the problem of non-participation and improve the results of digital health research accordingly.

## Methods

We developed the research approach of NPART (Evaluation of Non-Participation in Digital Health Research) for measuring and addressing non-participation of older people in digital health research, within the Supporting Self-Care by Information and Communication Technology (ICT) for Older People with Long-Term Conditions (ICT4Self-care) research programme (2015–2018) funded by the Swedish National Science Council and the Swedish Research Council for Health, Working Life and Welfare (VR-FORTE) (ref. 2014–4100).

### The NPART research approach

Non-participation is conceptualised as consisting of non-recruitment and self-selection processes (Fig. 1), and is studied in two phases of data collection. Following the two phases of data collection, comparisons between non-recruited, decliners and participants are conducted. Such an investigation based on hospital and survey data serves as a key basis for implementing improved sampling strategies, for example focused recruitment aiming at reaching specific underrepresented groups [54], and/or for conducting post hoc adjustments of the research outcomes, for example weighting outcomes according to the information on non-participation [55].

In a first phase, data collection must focus on the population at the recruitment site that has been defined as the target for the research, and which thus includes both the older people who will participate and those who will not due to non-recruitment or self-selection. Data consists of hospital or further registry information in combination with a recruitment log. Information based on hospital data allows for comparisons between the non-recruited, the decliners and the participants.

In a second phase, data collection must focus on those individuals who were deemed eligible for the research and were thus invited to participate. Therefore, those who will subsequently both consent and decline are approached. Data must consist of information on key aspects associated with the individual decision to participate. Data is collected through a survey questionnaire and combined with the information on (non-) participation from the recruitment log and with the reasons for declining, which could be collected as free responses during the recruitment process.

Information from the survey questionnaire allows for a more detailed comparison between decliners and participants, and for an interpretation of the decision on whether participate in the research.

To conduct this second phase of data collection, we developed an instrument – the NPART survey questionnaire – to explore key determinants of self-selection by including factors which are known to be associated with preparedness to participate in health-related research. In order to increase the response rate among the decliner group, the survey questionnaire should consist of a limited number of easy-to-answer questions, as recommended by the literature on non-response instruments [56, 57]. The NPART survey questionnaire consists of a limited number of short question items with a restricted number of response items.

The goal of the pilot study is to verify the construct validity [58, 59] of the questions used in the NPART survey questionnaire, namely the ability to cover the thematic dimensions of non-participation necessary in order to understand self-selection in digital health research.

### Designing the NPART survey questionnaire

Thematic areas for the survey questionnaire are based on a review of the literature. We focused on predictors for individual decisions to participate in digital health research, resulting in five thematic areas: socioeconomic factors, subjective health and subjective overall quality of life (subjective overall QOL), social participation, time resources, and use of technology and digital skills.

The NPART survey questionnaire consists of 36 questions (see Additional file 1). The questions for the NPART survey questionnaire were retrieved from major ageing studies and existing survey questionnaires. Some question items were adapted or developed. The main sources were the survey instrument of the Survey of Health, Ageing and Retirement in Europe (SHARE) [60], the Minimum European Health Module (MEHM) [61] and the World Health Organization Quality-of-Life Scale (WHOQOL-BREF) [62].

One additional question was included only for the purpose of the pilot study. This investigates preparedness to participate, namely the possible interest in participating in digital health research. Hence, it provides direct information on the individual decision: ‘If you received an invitation to participate in research testing a new technology that allows you to access healthcare services by phone, tablet or computer, would you like to participate in this research?’, with the response options ‘Yes’ and ‘No’. Such a question served only for the purpose of the pilot study to discriminate between participants and non-participants based on their preparedness to participate. Therefore, it is not intended to investigate reasons for participation or non-participation.

The socioeconomic factors were investigated with seven questions on age, gender, education (‘What is the highest level of education you have completed?’), job position (‘What is/was your primary job?’, ‘Which of the following categories best describes your primary area of employment?’), marital status (‘Do you have a partner?’) and living alone (‘Do you live alone?’).

Health and subjective overall QOL are covered by five questions investigating self-rated health (‘How is your health in general? It is’), health limitations (‘During the last 6 months, to what extent have you been limited because of your health in activities people usually do? Would you say you have been’), memory (‘How would you rate your memory at the present time? Would you say it is’), subjective overall QOL (‘How would you rate your quality of life?’) and self-reported need for support (‘Do you require any help taking care of your health, such as taking medications or attending/booking medical appointments?’).

Social participation is measured by four questions on involvement in social activities (‘During the past 12 months, how often have you participated in social activities such as volunteer or charity work, attended a training course, visited a sports club, social club or other kind of club, participated in the activities of a religious organisation, or participated in the activities of a political or community-related organisation?’), contact with children (if relevant) (‘During the past 12 months, how often did you have contact with your children, either in person, by phone, by mail, by e-mail or by any other electronic means?’, with response options including ‘I do not have any children’), contact with friends (‘During the past 12 months, how often did you have contact with or meet your friend and/or neighbours?’), and appropriateness of the amount of social contact (‘In the past 12 months, would you like to have had more contact with or met more frequently your children, relatives and/or friends?’).

Questions on time resources was measured by job hours (two questions: ‘Are you currently employed? If yes, how many hours per week?’) and caregiving (one question: ‘How often in the past 12 months did you care for a sick or disabled person?’).

Technology-related aspects were studied through questions investigating digital skills (ten questions: ‘How well do you think you master the following activities? Sending/receiving emails / Buying goods or services over the Internet / Reading or downloading online news, newspaper or magazines / Internet banking / Accessing institutions / Playing or downloading games, images, films or music / Listening to web radio or watching web television / Telephoning or making video calls over the Internet / Social networking, for example Facebook or Twitter / Posting messages to chat sites, blogs or forums, or instant messaging’), use of digital technologies (four questions: ‘Finally, we would like to ask you how often you use: a computer / a mobile phone / a smartphone and/or a tablet / a smart television and/or a games console’), perceived usefulness of digital health (three questions: ‘Do you think using a mobile phone, smartphone, tablet or the Internet might … Support you in performing everyday activities / Be useful in monitoring your health / Be useful for contacting nurses, physicians and other healthcare professionals’). A blank space for respondents to comment was also included.

The English version of the survey questionnaire was the master version. Regarding the development of the Swedish version, some of the items retrieved from existing survey questionnaires were already available in Swedish. If not, the English items were translated into Swedish by a native speaker.

All categorical variables that were not originally binary were re-coded as binary variables (e.g., ‘high/low’, ‘poor/good’), except for job position (four categories: ‘workers’, ‘clerks’, ‘self-employed’, ‘retired/no job’) and job hours (three categories: ‘no job/retired’, ‘up to 38 hours per week’, ‘39 or more hours per week’). As the goal of the pilot study was to understand the ability of the survey instrument to discriminate between decliner and consenting groups, the categories of the variables (e.g., ‘high/low’, ‘poor/good’) were determined in such a way as to maximise the differences between decliner and consenting people.

Education was coded as up to nine years (‘low’) and ten years or more (‘high’). Self-rated health was categorised as ‘poor’ (corresponding to the response items ‘fair’, ‘bad’ and ‘very bad’) and ‘good’ (corresponding to the response items ‘very good’ and ‘good’).

Regarding health limitations, we used the categories ‘having health limitations’ (which included those who answered that they were ‘severely limited’ or ‘limited but not severely’) and ‘not having limitations’ (which covered those people who answered ‘not limited at all’). Subjective overall QOL was re-coded as ‘low’, which corresponded to the answers ‘poor’ and ‘very poor’ or ‘neither poor nor good’, and ‘high’ which referred to reporting ‘very good’ or ‘good’ subjective overall QOL. Also, self-perceived memory performance was re-coded as ‘poor’ (for the answers ‘fair’, ‘bad’ and ‘very bad’ memory) and ‘good’ (for the answers ‘good’ and ‘very good’ memory). The self-reported need for support was expressed as occurring ‘often/sometimes’ (i.e., ‘almost daily’, ‘almost every week’ or ‘almost every month’) or ‘rarely/never’ (i.e., less often).

Social participation was described as ‘low’ and ‘high’, where the former corresponded to participating in various activities less often than monthly (response item: ‘less often’) and the latter referred to taking part in social activities ‘almost daily’, ‘almost every week’ or ‘almost every month’. Contact with children was described as occurring with ‘high frequency’ (which corresponded to the response items ‘almost daily’, ‘almost every week’ and ‘almost every month’) or ‘low frequency’ (i.e., ‘less often’ than every month). The variable ‘contact with friends’ was re-coded to ‘low frequency’, which referred to having contact ‘almost every month’ or ‘less often’ than every month, and ‘high frequency’, which corresponded to staying in contact with friends ‘almost daily’ or ‘almost every week’. The appropriateness of social contact was categorised as ‘I want more social contact’, which referred to wishing for ‘much more’ or ‘a little more’ contact, and ‘I want less/I do not want more contact’. The informal caregiving provided was re-coded as being provided ‘never/rarely’ or ‘often’.

Regarding the questions with sub-items, we added together the sub-items and summarised them into two categories (i.e., ‘low’ and ‘high’). The highest score for self-assessed digital skills was 50 (i.e., if respondents rated their skills as ‘excellent’ in all the activities) and the lowest was 10 (i.e., if the respondents rated their skills as ‘very poor’ in all the activities). We re-coded self-assessed digital skills as ‘low’ if the respondent scored between 10 and 37 overall and as ‘high’ if the respondent scored between 38 and 50 overall. The total score for the question on the use of digital technologies ranged between 4 and 20. ‘Low’ use of digital technologies corresponded to a score which ranged between 4 and 13, whereas ‘high’ use ranged between 14 and 20. Finally, respondents could score between 3 and 9 for the question on the perceived usefulness of digital technologies. We re-coded the perceived usefulness of digital technologies as ‘high’ for a total score between 7 and 9, and ‘low’ for a total score between 3 and 6.

The continuous variable ‘age’ was summarised by mean and standard deviation; all categorical variables were summarised by frequency and percentage.

### Piloting the NPART survey questionnaire

The NPART survey questionnaire was pre-tested with two study persons aged 68 and 61 in order to test the readability of the questions and to estimate the time needed for completion. After that, a pilot study was carried out with 70 older study persons (aged 60 or older) recruited at a day surgery unit at the Motala Hospital in Sweden. Study persons were approached during a recruitment process for a trial study within the ICT4Self-Care programme.

The average age of the participants was 68.8 years (SD = 6.2). Over half of the respondents (*n* = 37) were male and 33 were female. Of the 70 individuals involved, 45 reported they would agree to participate in research testing digital health technologies, while 25 of them would not.

### Analysis

The distribution of variables was compared with respect to preparedness to participate. For some questions we expected differences in the individual preparedness to participate, while for other questions we did not (e.g., gender, marital status, living alone).

To determine whether there was an association between preparedness to participate and the categorical variables investigated, the Chi-square test (χ^2^) or Fisher’s exact test were used depending on the expected frequencies in any of the cells of the contingency tables for a given variable. Fisher’s exact test was preferred if the frequency for any of the cells for a given variable was expected to be lower than five. Student’s t-test was used for continuous variables (i.e. age).

All analyses were performed using Stata v.14 [63].

## Results

The pre-test of the survey questionnaire suggested that the questions were all well readable. The survey questionnaire took approximately 10 to 15 min for the two participants to complete.

### Socioeconomic factors

In accordance with the literature, people who would agree to participate (i.e., the consenting group) in digital health research were on average slightly younger than those who would not agree to participate (i.e., the decliner group), with a mean age = 69.6, SD = 6.1 and a mean age = 68.3, SD = 6.2 respectively, and had a higher level of education (27 out of 44 respondents) compared with the decliner group (10 out of 25), although the differences were not significant (Table 1). We did not expect any associations between gender, job position, marital status or living alone and preparedness to participate.

**Table 1:** NPART questions on socioeconomic factors by preparedness to participate or not in digital health research

			Preparedness to participate		Chi^2^ / t value	*p*-value^a^
		Total sample n (valid column %)	Decliner group n (valid column %)	Consenting group n (valid column %)		
Education	Low education	32 (46)	15 (60)	17 (39)		
	High education	37 (54)	10 (40)	27 (61)	2.92	*0.087*
	*Missing*	*1*	*0*	*1*		
Age (mean (SD))		68.8 (6.2)	69.6 (6.1)	68.3 (6.2)	0.79	*0.430*
Job position	Workers	25 (36)	11 (44)	14 (31)		
	Clerks	26 (37)	8 (32)	18 (40)		
	Self-employed	12 (17)	4 (16)	8 (18)		
	Retired/no job	7 (10)	2 (8)	5 (11)	–	*0.785*^*a*^
	*Missing*	*0*	*0*	*0*		
Gender	Women	33 (47)	12 (48)	21 (47)		
	Men	37 (53)	13 (52)	24 (53)	0.01	*0.91*
	*Missing*	*0*	*0*	*0*		
Partner	Yes	57 (83)	19 (79)	38 (84)		
	No	12 (17)	5 (21)	7 (16)	–	*0.740*^*a*^
	*Missing*	*1*	*1*	*0*		
Living alone	Yes	55 (82)	18 (82)	37 (82)		
	No	12 (18)	4 (18)	8 (18)	–	*1.000*^*a*^
	*Missing*	*3*	*3*	*0*		
Total (*n* = 70)		70	25 (36)	45 (64)		

^a^The *p*-value generally refers to the Chi^2^ statistics, but Fisher’s exact test was performed if frequencies smaller than 5 were expected in any of the cells for a given variable

### Health and subjective overall QOL

In line with the relevant literature, people in the decliner group self-rated their memory as poorer (11 out of 24) in comparison with the consenting group (7 out of 45), χ^2^ (1, *n* = 69) = 7.44, *p* = 0.006 (Table 2).

**Table 2:** NPART questions on health-related aspects and subjective overall QOL, by preparedness to participate or not in digital health research

			Preparedness to participate		Chi^2^	*p*-value^a^
		Total sample n (valid percent %)	Decliner group n (valid percent %)	Consenting group n (valid percent %)		
Self-rated health	Poor	12 (17)	6 (25)	6 (13)		
	Good	57 (83)	18 (75)	39 (87)	–	*0.318*^a^
	*Missing*	*1*	*1*	*0*		
Health limitations	Limitations	45 (65)	18 (75)	27 (60)		
	No limitations	24 (35)	6 (25)	18 (40)	1.55	*0.213*
	*Missing*	*1*	*1*	*0*		
Subjective overall QOL	Low	8 (12)	4 (17)	4 (9)		
	High	61 (88)	20 (83)	41 (91)	–	*0.435*^a^
	*Missing*	*1*	*1*	*0*		
Self-perceived memory performance	Poor	18 (26)	11 (46)	7 (16)		
	Good	51 (74)	13 (54)	38 (84)	7.44	*0.006*
	*Missing*	*1*	*1*	*0*		
Self-reported need for support	Rarely/never	64 (93)	23 (96)	41 (91)		
	Often/sometimes	5 (7)	1 (4)	4 (9)	–	*0.652*^a^
	*Missing*	*1*	*1*	*0*		
Total (n = 70)		70	25 (36)	45 (64)		

^a^The *p*-value generally refers to the Chi^2^ statistics, but Fisher’s exact test was performed if frequencies smaller than 5 were expected in any of the cells for a given variable

Also, respondents in the decliner group had poorer self-rated health (6 out of 24) and more health limitations (18 out of 24) than those who would agree to participate (6 out of 39 and 27 out of 45 respectively), even though such frequencies were not significantly different. In line with previous studies, older people in the consenting group tended to show higher subjective overall QOL (41 out of 45) than those in the decliner group (20 out of 24).

In contrast to what we expected, the item on self-reported need for support did not clearly discriminate between the two groups. This suggests that this item might need to be reconsidered.

### Social participation

We found that people in the consenting group had higher social participation (25 out of 44) compared to those who would not agree to participate in digital health research (7 out of 24), χ^2^ (1, *n* = 68) = 4.76, *p* = 0.029 (Table 3). Consenting individuals also had more contact with their children and friends than the decliner group did, although the frequencies were not significantly different. The consenting group expressed a desire for more social contact with family and friends (22 out of 43) compared with the respondents in the decliner group (6 out of 23), χ^2^ (1, *n* = 67) = 4.33, *p* < 0.05.

**Table 3:** NPART questions on social participation by preparedness to participate or not in digital health research

			Preparedness to participate		Chi^2^	*p*-value^a^
		Total sample n (valid column %)	Decliner group n (valid column %)	Consenting group n (valid column %)		
Social participation	Low frequency	36 (53)	17 (71)	19 (43)		
	High frequency	32 (47)	7 (29)	25 (57)	4.76	*0.029*
	*Missing*	*2*	*1*	*1*		
Contact with children	Low frequency	8 (12)	4 (17)	4 (9)		
	High frequency	61 (88)	20 (83)	41 (91)	–	*0.435*^a^
	*Missing*	*2*	*1*	*1*		
Contact with friends	Low frequency	7 (10)	4 (17)	3 (7)		
	High frequency	62 (90)	20 (83)	42 (93)	–	*0.227*^a^
	*Missing*	*1*	*1*	*0*		
Appropriateness of amount of social contact	I want more social contact	28 (42)	6 (25)	22 (51)		
	I want less/I do not want more social contact	39 (58)	18 (75)	21 (49)	4.33	*0.037*
	*Missing*	*3*	*1*	*2*		
Total (n = 70)		70	25 (36)	45 (64)		

^a^The *p*-value generally refers to the Chi^2^ statistics, but Fisher’s exact test was performed if frequencies smaller than 5 were expected in any of the cells for a given variable

### Time resources

People in the decliner group provided slightly more informal care (5 out of 22) compared to the other group (6 out of 43), although this difference was not statistically significant. However, this result must be interpreted with caution since we discovered that the wording in the item was to some extent misleading. In particular, it was not clear that the item was about informal care and did not include formal care. Hence, we cannot interpret answers which relate to respondents working in healthcare occupations. In contrast to our expectations, we found no effect of working hours on preparedness to participate (Table 4).

**Table 4:** NPART questions on time resources by preparedness to participate or not in digital health research

			Preparedness to participate		Chi^2^	*p*-value^a^
		Total sample n (valid column %)	Decliner group n (valid column %)	Consenting group n (valid column %)		
Job hours	No job/retired	50 (74)	18 (75)	32 (73)		
	< 39 h	7 (10)	2 (8)	5 (11)		
	≥ 39 h	11 (16)	4 (17)	7 (16)	–	*1.000*^*a*^
	*Missing*	*2*	*1*	*1*		
Informal caregiving	Never/rarely	54 (83)	17 (77)	37 (86)		
	Often	11 (17)	5 (23)	6 (14)	–	*0.487*^*a*^
	*Missing*	*5*	*3*	*2*		
Total (n = 70)		70	25 (36)	45 (64)		

^a^The *p*-value generally refers to the Chi^2^ statistics, but Fisher’s exact test was performed if frequencies smaller than 5 were expected in any of the cells for a given variable

### Digital skills, use of digital technologies and perceived usefulness of digital technologies

In accordance with the literature, respondents in the consenting group showed higher digital skills (24 out of 42) compared to those in the decliner group (6 out of 21), χ^2^ (1, *n* = 63) = 4.58, *p* = 0.032, and higher use of digital technologies (33 out of 41) than the respondents who would decline to participate in digital health research (11 out of 20), χ^2^ (1, *n* = 61) = 4.34, *p* = 0.037 (Table 5). Also, although the frequencies did not differ significantly, the respondents in the consenting group indicated a higher perceived usefulness of digital technologies (28 out of 45) compared to the people in the decliner group (9 out of 24).

**Table 5:** NPART questions on technology-related aspects by preparedness to participate or not in digital health research

			Preparedness to participate		Chi^2^	*p*-value^a^
		Total sample n (valid column %)	Decliner group n (valid percent %)	Consenting group n (valid percent %)		
Self-assessed digital skills	Low	33 (52)	15 (71)	18 (43)		
	High	30 (48)	6 (29)	24 (57)	4.58	*0.032*
	*Missing*	*7*	*4*	*3*		
Use of digital technologies	Low	17 (28)	9 (45)	8 (19)		
	High	44 (72)	11 (55)	33 (81)	4.34	*0.037*
	*Missing*	*9*	*5*	*4*		
Perceived usefulness of digital technologies	Low	32 (46)	15 (63)	17 (38)		
	High	37 (54)	9 (37)	28 (62)	3.85	*0.050*
	*Missing*	*1*	*1*	*0*		
Total (*n* = 70)		70	25 (36)	45 (64)		

^a^The *p*-value generally refers to the Chi^2^ statistics, but Fisher’s exact test was performed if frequencies smaller than 5 were expected in any of the cells for a given variable

### Summary of the results

In accordance with previous studies [31, 34], people in the consenting group were on average slightly younger and more highly educated compared to the decliners.

The questions on subjective health and subjective overall QOL discriminated between people in the decliner and consenting groups, especially in terms of self-perceived memory performance. People in the consenting group reported having a better memory than the decliners, which is in line with the relevant literature suggesting that individuals with cognitive impairments are less often represented in health-related studies [32, 36].

We found an association between preparedness to participate and aspects related to social participation. People in the consenting group said that they participated more in social activities and wanted more social contact than they currently have, compared to the people in the decliner group.

As suggested by previous studies [33, 52], consenting and decliner people differed regarding technology-related aspects. People in the consenting group reported having better digital skills and using digital technologies more often, compared to the decliners.

Some of the determinants investigated by the survey questionnaire did not clearly discriminate between the consenting and decliner groups (e.g., age, education, health status, health limitations). Nevertheless, we could observe differences between the two groups which pointed in the expected directions. The absence of clearer effects is presumably due to the nature and the size of the sample.

## Discussion

The overall aim of this paper was to introduce, discuss and test an instrument designed to measure non-participation in digital health research. Such an instrument would serve to generate information which is needed for implementing further sampling strategies and/or post hoc adjustments of outcomes produced from biased samples.

First, we examined the nature of non-participation and its importance in digital health research. Second, we discussed the conceptualisation of an approach to study non-participation and the two processes behind it – non-recruitment and self-selection. Based on that, we introduced an instrument for analysing self-selection and then presented the results of a pilot study.

The process of non-recruitment can be analysed using hospital or further registry data. Such hospital data is usually quite limited in terms of indicators, and we need to add survey data from those who may prefer not to participate to understand the full process of non-participation thoroughly. This means developing a survey instrument that allows self-selection to be described with a non-response study approach.

In line with the theoretical assumptions on the self-selection process, the results from the pilot study of the survey questionnaire show that the instrument is sensitive to the differences between participants and non-participants, even if we only use short and simple indicators for the short non-response instrument. Compared with decliners, people in the consenting group were on average slightly younger and more educated. They reported having better memory, higher social participation, better skills and to be more frequent users of digital technologies. This survey questionnaire can be used successfully to analyse the self-selection of older people and is suggested for further use in digital health research.

As an instrument for the analysis of self-selection, the survey questionnaire must be viewed in combination with the reasons collected for declining. Overall, such a combination of data allows for the interpretation of the individual decision to participate in a study testing new digital health technologies. In the broader context of addressing non-participation in digital health research, the survey instrument provides researchers with the information needed to implement improved sampling strategies and/or outcome corrections.

The information generated by the NPART research approach can guide focused recruitment among underrepresented groups, for example those with lower digital skills or cognitive impairments. In addition, information based on the NPART research approach is crucial for performing post hoc corrections to the study outcomes by, for example, weighting procedures that give different weights to different groups in the sample according to the proportion they represent in the total target population of interest. In sum, by allowing such strategies, data generated by the NPART research approach and its questionnaire prevents overestimating and underestimating the effects of an intervention on a target population and improves research results.

This investigation is not without limitations. The pilot study was conducted on a small non-random sample and we believe this prevented us from finding stronger associations between non-participation and key aspects investigated. Nonetheless, the directions of the results were overall in line with the relevant literature on non-participation.

As a short non-response instrument, the NPART survey questionnaire investigates only a selection of aspects for each thematic area. Therefore, some detailed information might be missed, such as details on specific health impairments. However, some of this information, e.g., hearing and sight impairments, dementia and cognitive impairments, might be accessed from hospital data or other registries.

The items that investigated time resources and the self-reported need for support should be reconsidered as they were not sufficiently sensitive to capture the differences between participants and non-participants, as expected. In connection with time availability, some relevant aspects were not covered in the questionnaire, e.g., taking care of grandchildren and children. This should also be re-examined. Moreover, as the item on informal caregiving does not clearly measure informal care, we cannot interpret the results from it.

Finally, it is likely that the NPART survey questionnaire will encounter non-response itself, especially from those people who are typically the hardest to reach. The results of an investigation on preparedness to participate would not be changed in such an eventuality but might show a smaller effect.

## Conclusions

The NPART survey questionnaire is valid for analysing individual preparedness to participate in digital health research along the lines of the relevant literature.

As a whole, the NPART research approach can be used to clarify and deepen the mechanisms of non-participation in the exclusion of people from the digital health research and to illuminate the differences between older study participants and older non-participants.

The NPART research approach and its survey instrument are flexible in their application. They can be embedded into a wide range of recruitment processes in digital health studies and target diverse populations.

Recruiting representative samples of people will always present a challenge. Some people are especially hard to reach and recruit in digital health research, such as older people and those who are less educated, less familiar with technological advances and less involved socially. These people are often excluded and underrepresented in research, even though they are targeted by the digitalisation process which is changing the provision of healthcare services. By studying the two steps of non-participation – non-recruitment and self-selection – and characterising participants and non-participants, strategies supporting needs recognition among underrepresented groups can be implemented to improve research outcomes of digital health research and better inform policy and practice.

## Supplementary information

**Additional file 1.** NPART survey questionnaire.

## Data Availability

The materials and datasets used and analysed in the current study are available from the corresponding author on request.

## Abbreviations

ICT: Information communication technology
MEHM: Minimum european health module
NPART: Evaluation of non-participation in digital health research
QOL: Quality of life
SHARE: Survey of health, ageing and retirement in Europe
WHOQOL-BREF: World health organization quality-of-life scale
