# Supplementary material for: A research tool for measuring non-participation of older people in research on digital health
Source: BMC Public Health. 2019 Nov 8;19:1487. doi: 10.1186/s12889-019-7830-x (PMC6842243; doi:10.1186/s12889-019-7830-x)
Supplement: Supplementary file 1 — Additional file 1. NPART survey questionnaire. [file 12889_2019_7830_MOESM1_ESM.pdf]

**1** Please indicate your **gender**:

| Male | Female |
|---|---|
| ☐2 | ☐1 |

**2** Please indicate your **birth** (yymmdd)?

[ ]

**3** What is the highest **level of education** you have completed?

- ☐1 Early childhood education
- ☐2 Primary education
- ☐3 Lower secondary education
- ☐4 Upper secondary education
- ☐5 Post-secondary non-tertiary education / Tertiary education
- ☐6 Short-cycle tertiary education
- ☐7 Bachelor's or equivalent level
- ☐8 Master's or equivalent level
- ☐9 Doctoral or equivalent level

**4** Are you currently **employed**?

| Yes | No |
|---|---|
| ☐1 | ☐0 |

*If **yes**, how many hours per week?*

[ ]

**5** What is/was your primary **job**?

[ ]

**6** Which of the following categories best describes your primary **area of employment**?

- ☐1 Manager
- ☐2 Professionals
- ☐3 Technicians and Associate Professionals
- ☐4 Clerical Support Workers
- ☐5 Services and Sales Workers
- ☐6 Skilled Agricultural, Forestry and Fishery Workers
- ☐7 Craft and Related Trades Workers
- ☐8 Plants and Machine Operators, and Assemblers
- ☐9 Elementary Occupations
- ☐10 Armed Forces Occupations
- ☐11 I have never worked

**7** Do you have a **partner**?

| Yes | No |
|---|---|
| ☐1 | ☐0 |

**8** Do you live **alone**?

| Yes | No |
|---|---|
| ☐1 | ☐0 |

**9** How often in the past 12 months did you **care for a sick or disabled person**?

| Almost daily | Almost every week | Almost every month | Less often |
|---|---|---|---|
| ☐4 | ☐3 | ☐2 | ☐1 |

**10** During the past 12 months, how often have you participated in **social activities** such as volunteer or charity work, attended a training course, visited a sports club, social club or other kind of club, participated in the activities of a religious organisation, or participated in the activities of a political or community-related organisation?

| Almost daily | Almost every week | Almost every month | Less often |
|---|---|---|---|
| ☐4 | ☐3 | ☐2 | ☐1 |

**11** During the past 12 months, how often did you **have contact with your children**, either in person, by phone, by mail, by e-mail or by any other electronic means?

| Almost daily | Almost every week | Almost every month | Less often | *I do not have any children* |
|---|---|---|---|---|
| ☐5 | ☐4 | ☐3 | ☐2 | ☐1 |

**12** During the past 12 months, how often did you have contact with or meet your **friend and/or neighbours**?

| Almost daily | Almost every week | Almost every month | Less often |
|---|---|---|---|
| ☐4 | ☐3 | ☐2 | ☐1 |

**13** In the past 12 months, would you like to have had more **contact with** or met more frequently your children, relatives and/or friends?

| Much more | Little more | Not more | Less |
|---|---|---|---|
| ☐4 | ☐3 | ☐2 | ☐1 |

**14** How would you rate **your memory** at the present time? Would you say it is…

| Very good | Good | Fair | Bad | Very bad |
|---|---|---|---|---|
| ☐5 | ☐4 | ☐3 | ☐2 | ☐1 |

**15** How is your **health** in general? It is…

| Very good | Good | Fair | Bad | Very bad |
|---|---|---|---|---|
| ☐5 | ☐4 | ☐3 | ☐2 | ☐1 |

**16** During the last 6 months, to what extent have you been **limited because of your health** in activities people usually do? Would you say you have been…

| Severely limited | Limited but not severely | Not limited at all |
|---|---|---|
| ☐3 | ☐2 | ☐1 |

**17** Do you require any help taking care of your health, such as taking medications or attending/booking medical appointments?

| Almost daily | Almost every week | Almost every month | Less often |
|---|---|---|---|
| ☐4 | ☐3 | ☐2 | ☐1 |

**18** How would you rate your **quality of life**?

| Very good | Good | Neither poor nor good | Poor | Very poor |
|---|---|---|---|---|
| ☐5 | ☐4 | ☐3 | ☐2 | ☐1 |

***Now there are some questions regarding the use of the internet and technology***

**19** How well do you think you master the following activities?

| | Excellent | Good | Fair | Poor | Very poor |
|---|---|---|---|---|---|
| Sending/receiving e-mails | ☐5 | ☐4 | ☐3 | ☐2 | ☐1 |
| Buying goods or services over the Internet | ☐5 | ☐4 | ☐3 | ☐2 | ☐1 |
| Reading or downloading online news, newspaper or magazines | ☐5 | ☐4 | ☐3 | ☐2 | ☐1 |
| Internet banking | ☐5 | ☐4 | ☐3 | ☐2 | ☐1 |
| Accessing institutions | ☐5 | ☐4 | ☐3 | ☐2 | ☐1 |
| Playing or downloading games, images, films or music | ☐5 | ☐4 | ☐3 | ☐2 | ☐1 |
| Listening to web radio or watching web television | ☐5 | ☐4 | ☐3 | ☐2 | ☐1 |
| Telephoning or making video calls over the internet | ☐5 | ☐4 | ☐3 | ☐2 | ☐1 |
| Social networking, for example Facebook or Twitter | ☐5 | ☐4 | ☐3 | ☐2 | ☐1 |
| Posting messages to chat sites, blogs or forums, or instant messaging | ☐5 | ☐4 | ☐3 | ☐2 | ☐1 |

**20** Do you think using a mobile phone, smartphone, tablet or the Internet might…

| | Yes | Maybe | No |
|---|---|---|---|
| **Support** you in performing everyday activities (e.g. remembering medications and appointments, calling for emergency) | ☐3 | ☐2 | ☐1 |
| Be useful in **monitoring** your health (e.g. sleeping, diet, blood pressure, general symptoms) | ☐3 | ☐2 | ☐1 |
| Be useful for contacting **nurses, physicians and other healthcare professionals** | ☐3 | ☐2 | ☐1 |

**21** Finally, we would like to ask you how often you use:

| | Daily | At least once per week | At least once per month | Less than once per month | Never |
|---|---|---|---|---|---|
| Computer | ☐5 | ☐4 | ☐3 | ☐2 | ☐1 |
| Mobile phone | ☐5 | ☐4 | ☐3 | ☐2 | ☐1 |
| Smartphone and/or tablet | ☐5 | ☐4 | ☐3 | ☐2 | ☐1 |
| Smart Television and/or games console | ☐5 | ☐4 | ☐3 | ☐2 | ☐1 |

**22** If you received an invitation to participate in a research testing a new technology that allows you to access healthcare services by phone, tablet or computer, would you like to participate in this research?

| Yes | No |
|---|---|
| ☐1 | ☐0 |

## Thank you for answering the questionnaire!

| Is there anything you want to add or comment on? Please write in the comments field below. |
|---|
| |
